# Enhancing Success in the ABO-Incompatible Kidney Transplantation: A Case Report

**DOI:** 10.7759/cureus.62350

**Published:** 2024-06-14

**Authors:** D. S. Rana, A. K. Bhalla, Ashwani Gupta, Manish Malik, Anurag Gupta

**Affiliations:** 1 Nephrology, Sir Ganga Ram Hospital, New Delhi, IND

**Keywords:** plasmapheresis, antibody titer, immunoadsorption column, abo incompatible kidney transplant, desensitization

## Abstract

Kidney transplantation is the preferred treatment for end-stage renal disease (ESRD); however, ABO incompatibility (ABOi) poses challenges due to increased graft rejection risk. Desensitization strategies, including immunoadsorption (IA), aim to overcome ABOi barriers. The objective of this case report was to present the initial findings and patient outcomes of ABOi kidney transplantation (KT) using two different brands of IA columns (Glycosorb^®^ ABO and SECORIM^®^-ABO) in reducing isoagglutinin titers to the desired target level.

We present a case report of a 51-year-old male with ESRD secondary to diabetic kidney disease who underwent desensitization for ABOi KT, involving rituximab administration followed by IA using Glycosorb^® ^and Vitrosorb SECORIM^®^-ABO columns and plasmapheresis (PP). Glycosorb^®^ ABO column decreased anti-B titers from an initial level of 1:128/1:128 to 1:64/1:64 (target range ≤1:8); however, the titers rebounded to 1:64 following the fourth session of PP. Subsequent use of Vitrosorb SECORIM^®^-ABO column achieved target titers of 1:4, enabling successful transplantation with satisfactory graft function. Monitoring included anti-B IgG/IgM titer levels post IA columns, IA column reuse, kidney function, and adverse events. The IA columns were well tolerated. Desensitization using IA columns effectively reduced anti-B titers, facilitating successful ABOi KT.

## Introduction

Globally, a significant number of individuals experience end-stage renal disease (ESRD), with approximately one-third meeting the criteria for kidney transplantation [1]. Kidney transplantation has emerged as the optimal therapeutic approach for individuals with stage 5 chronic kidney disease, offering substantial advantages in terms of long-term survival as compared to long-term dialysis. In India, the primary source of organs for transplantation often comes from living-related kidney donors within the immediate family. The potential success of kidney transplantation is constrained by ABO incompatibility (ABOi) and histo-incompatibility. Advances in the twenty-first century have enabled organ transplantations across donors/recipients with antibody incompatibility, providing survival and quality of life advantages as compared to individuals remaining on the waiting list and undergoing repeated dialysis [2].

Circulating antibodies generated in response to ABO antigens absent in the recipient can lead to graft rejection through an antibody-mediated process, also known as antibody-mediated rejection (AMR). Therefore, it is necessary to eliminate these antibodies before transplantation [3]. Various methods have been employed to eliminate ABO blood group antibodies, including double-filtration plasma exchange, routine therapeutic plasma exchange, nonspecific and specific immunoadsorption (IA, proteasome inhibitors), anti-CD20 and CD38 monoclonal antibodies, cysteine protease, and interleukin-6 inhibitors [4,5]. Different approaches to desensitization vary across centers and are based on two combined approaches [1,6-8]. The first approach encompasses immunosuppression, utilizing steroids, tacrolimus, and mycophenolic acid alongside rituximab. The second approach involves apheresis, a procedure designed to eliminate isoagglutinins. Apheresis can be conducted through plasmapheresis (PP) or double-filtration plasmapheresis (DFPP). While PP is effective, it is associated with the removal of clotting factors, posing a risk of severe bleeding during the peri-transplantation period [1,8,9]. With high antibody titer, selective IA is preferred, thus avoiding side effects associated with PP. These strategies have contributed to positive outcomes for both grafts and patients in the short and long terms [2]. In developing countries like India, where transplant choices are restricted, ABOi stands as a significant constraint, particularly for living donors, resulting in the rejection of approximately 35% of living kidney donors. The primary objective is to reduce anti-ABO antibody titers before surgery, and many centers follow the guidelines that titers should be ≤1:8 before transplantation. This limit is established based on empirical evidence [10].

Over the past two decades, there has been a progression in ABOi transplantation protocols, shifting from splenectomy to targeted removal of specific antibodies through IA techniques. ABOi kidney transplantation (ABOi KT) remains infrequent in developing nations owing to challenges such as elevated costs, limited experience and infrastructure, and the potential for AMR. There is global variation in ABOi transplantation protocols concerning antibody removal techniques, accepted and targeted antibody titers, methods of antibody detection, and maintenance of immunosuppression. In Europe, many preconditioning protocols predominantly employ IA techniques using antigen-specific columns. Among these columns are two commercially available types, including the single-use Glycosorb® ABO column (Glycorex AB, Lund, Sweden) and the reusable ABO Adsopak® column [11]. Additionally, a third column named SECORIM®-ABO has been introduced too [12]. The IA protocol has garnered widespread acceptance and has been effectively executed in numerous reputable institutions globally [11-13].

IA is the most specific, targeting only anti-blood-group antibodies. However, utilizing the column only once significantly increases the overall cost of the transplant procedure [14]. This aspect holds greater significance in developing nations, such as India, where patients are responsible for covering the expenses associated with transplantation. A study by Schiesser et al. investigated the effectiveness and safety of reusing IA columns in ABOi KT. The findings of this study support the reusability of IA columns, making them commercially sustainable [4,14]. A prospective study by Mukherjee et al. concluded that the use of IA columns efficiently depleted antibody titers and that the outcomes of ABOi renal transplantation were satisfactory [11]. A case series conducted by Kalra included 10 patients with ESRD who required desensitization for ABOi living donor KT. The investigator used the SECORIM®-ABO column from Vitrosorb AB, Malmö, Sweden to reduce ABO-isoagglutinin titers in all prospective ABOi KT recipients during desensitization. Kalra demonstrated the effectiveness of the IA column, where three sessions of IA successfully decreased the titer level to 1:4 in a patient with a baseline isoagglutinin IgG titer of 1:512. Additionally, there were no instances of bleeding-related complications after the procedure or postoperatively among the patients [12]. This targeted elimination of antibodies through the IA column prevents various adverse effects, in contrast to alternative pretreatment approaches such as plasma exchange. The precise removal of antibodies by the IA column ensures the preservation of essential physiological plasma components, including coagulation factors. Additionally, the IA columns present a significant advantage over alternative methods [15].

In clinical practice, data on kidney transplantation using IA columns are limited. The objective of this case report was to present the initial findings and patient outcomes of ABOi KT utilizing two different brands of IA columns (Glycosorb® ABO and SECORIM®-ABO) to highlight their effectiveness in reducing isoagglutinin titers to the desired target level.

## Case presentation

A 51-year-old male with a medical history of diabetes mellitus, hypertension, and stage 5 chronic kidney disease (undergoing maintenance hemodialysis twice a week) underwent an assessment of his suitability for a renal allograft. On the day of admission (day -22), the patient's physical examination revealed a pulse rate of 104/min, blood pressure of 180/110 mmHg, and a temperature of 37°C. The patient had a residual urine output of 250-300 mL/day and cardiomyopathy, with a left ventricular ejection fraction of 30%. The potential kidney donor was his wife, whose blood group was B-positive, while the recipient had O-positive blood. Human leukocyte antigen matching revealed a 2/6 match.

To prepare for renal transplantation, the patient underwent a desensitization protocol, starting with the administration of inj. rituximab 200 mg on the day (-42). The baseline anti-B titers for immunoglobulin (Ig) IgG/IgM were 1:128/1:128. The patient was initially treated with an IA column (Glycosorb®). Two Fresenius 4008S dialysis machines (Fresenius Medical Care, Bad Homburg, Germany) were utilized for IA. One P2 dry plasma filter was used to separate blood cells from the plasma, and initially, the blood flow rate was kept at 80 mL/minute and later was gradually increased to 200 mL/minute; subsequently, the plasma flow rate was 20 mL/minute which gradually reached 50 mL/minute. The plasma was then passed through a Glycosorb® column and reinfused back into the patient.

The initial intervention involved the use of an IA column, Glycosorb®, after rituximab administration. The use of the Glycosorb® first column initially reduced the anti-B titer levels to 1:64 from 1:128. However, since no further reduction in the anti-B titer levels could be achieved even after repeated three IA sessions with the Glycosorb® first column, another fresh Glycosorb® column was used for the subsequent three sessions, and only one titer reduction could be achieved. The resultant titer was stagnant at 1:64. Next, four PP sessions were conducted. Consequently, the anti-B titer was reduced to 1:16/1:32. Nonetheless, the titer level bounced back at 1:64 after PP session four. This sudden increase in titer after the fourth PP is attributed to the fact that PP involves fluid replacements, which can lead to a rise in the titer. As the anti-B titer did not reach the targeted levels, the physicians decided to use IA with another brand, the SECORIM®-ABO column from Vitrosorb AB, Malmö, Sweden, resulting in a titer value of an acceptable range of 1:4 (Table 1).

**Table 1:** Anti-B IgG/IgM titer levels post-use of IA columns and PP sessions The initial anti-B titer (IgG/IgM) level of the patient was 1:128/1:128. IA: immunoadsorption; IgG: immunoglobulin G; IgM: immunoglobulin M; PP: plasmapheresis

Modality	Days	Anti-B IgM/IgG titer levels post IA column and PP session
Glycosorb I (ABO Column)	Day -22	1:64/1:64
Glycosorb I (reuse)	Day -21	1:64/1:64
Glycosorb I (reuse)	Day -19	1:64/1:64
Glycosorb II (ABO Column)	Day -15	1:32/1:64
Glycosorb II (reuse)	Day -14	1:32/1:64
Glycosorb II (reuse)	Day -13	1:32/1:64
PP 1	Day -12	1:16/1:32
PP 2	Day -11	1:16/1:32
PP 3	Day -7	1:32/1:64
PP 4	Day -6	1:64/1:64
Vitrosorb (ABO Column)	Day -2	1:16/1:16
Vitrosorb (ABO Column)	Day -1	1:4/1:4
Day of transplantation	Day 0	

Immuno-suppressants tacrolimus/mycophenolate mofetil were started according to body weight on day -2 and Solu-Medrol on day -1. The patient underwent successful kidney transplant surgery (on day 0). The first dose of antithymocyte globulin (ATG) 50 mg was administered on the morning of the day of surgery. The second and third doses of ATG 50 mg were administered on postoperative days 1 and 2, respectively. The intraoperative and immediate postoperative periods were uneventful. The immediate postoperative diuresis was brisk and profuse. A kidney graft ultrasound was performed on day 5, revealing a fluid collection measuring 7.4 x 5.3 x 4.2 cm (volume 88 mL) in the anterolateral region and 4.0 x 3.2 x 3.2 cm (volume 22 mL) in the inferomedial area of the transplanted kidney. Kidney perfusion was deemed satisfactory with normal flow.

The anti-B titers were measured daily post-transplantation for 10 days, and no increase in titers was observed; they were found to be constant for IgG at 1:8 (Table 2).

**Table 2:** Anti-B IgM/IgG titers monitoring post renal transplantation IgG: immunoglobin G; IgM: immunoglobin M

Days	Anti-B IgM/IgG titer
Day 1	1:8/1:8
Day 2	1:8/1:8
Day 3	1:16/1:8
Day 4	1:16/1:16
Day 5	1:8/1:8
Day 6	1:8/1:8
Day 7	1:8/1:8
Day 8	1:8/1:8
Day 9	1:8/1:8
Day 10	1:4/1:8

The patient remained stable, and graft recovery was favorable, with serum creatinine decreasing to 0.78 mg/d. The patient was hemodynamically stable and discharged on day 10 with a creatinine level of 1 mg/dL.

## Discussion

An enhanced understanding of immunopathogenesis and aggressive immunosuppression has led to the development of effective desensitization protocols for ABOi KT. Current ABOi KT strategies involve pre-transplant antibody removal utilizing PP or IA alongside the robust maintenance of immunosuppressives (e.g., tacrolimus and mycophenolate mofetil) to prevent rejection. The challenges include desensitizing high anti-ABO antibody producers and managing the increased risk of infection and malignancy associated with intensified immunosuppression. Optimizing desensitization and tailoring immunosuppression for individual patients is crucial for improving ABOi KT outcomes [16]. In our case, desensitization commenced with the initial use of the IA column (Glycosorb® ABO column), followed by PP, which reduced the titer to 1:16/1:32. However, the significant increase in antibody titer (IgM/IgG, 1:64/1:64) observed as a rebound after the fourth PP session could be due to the proliferation of antibody-producing cells, an increase in the synthetic activity of these cells, or the fluid replacement aspect of PP [17]. Subsequently, we managed to effectively reduce the anti-B titer to the desired level through two additional sessions with a different brand of IA column (SECORIM®-ABO).

Lowering the concentration of isoagglutinins is crucial to minimize the likelihood and intensity of AMR episodes. The inclusion of rituximab in the treatment plan necessitates its combination with PP or IA, as the majority of plasma cells do not express CD20, ensuring the effective elimination of isoagglutinins [18]. Our approach also involved the use of rituximab followed by IA procedure by utilizing columns such as Glycosorb® and SECORIM®-ABO columns and eventually administering immunosuppressives such as tacrolimus/mycophenolate mofetil and ATG to prevent the occurrence of an AMR episode in our patient.

The development of novel blood group-specific IA columns has facilitated a more efficient and rapid approach to the desensitization of living donor ABOi KT. The benefits of employing IA for desensitization include its specificity against blood group antibodies, shorter duration of desensitization, more significant plasma volume processing, and the ability to achieve the desired reduction in titers without the need for replacement fluid [12]. Therefore, perioperative complications, length of hospital stay, and related costs were reduced. In this case study, the authors processed large plasma volumes per IA procedure and reused each column up to three times. This translated into a shorter period of desensitization, earlier surgery, and a shorter hospital stay for the patient.

The IA approach has gained popularity over time and has been successfully implemented in numerous established medical centers worldwide. Patients with elevated baseline titers are commonly advised to undergo treatment with an IA column because of their specificity and effectiveness. Given the high cost of a single IA column and its limited ability to consistently reach the targeted anti-ABO titers in a single use, its widespread adoption is limited, and it is often reused more than once. The practice of reusing IA columns has been reported in Switzerland and in India. Nevertheless, the high cost associated with IA columns restricts their practicality in less-established centers, particularly those in developing and underdeveloped countries. The authors attempted to address this limitation by reusing the column despite the manufacturer's recommendation for single use. This approach effectively lowered the titers and reduced the cost to the patient and his family. Adhering to the manufacturers' instructions and prioritizing IA use in patients with high antibody titers (≥256) would be advisable if the cost was not a concern [4,15]. The adopted approach underpins the positive outcomes of patients' quality of life, too.

In a case series conducted by Tiwari et al., 16 IA procedures were performed on five patients in a tertiary care center in India. All the patients underwent successful ABOi KT. No adverse events were reported during the IA procedure. Furthermore, all patients underwent successful desensitization before IA and continued to perform well clinically, with a mean follow-up period of 8.8 months [19]. In this case, successful kidney transplant surgery resulted in an uneventful intraoperative and immediate postoperative period. The immediate postoperative period was uneventful, and kidney perfusion displayed satisfactory results, exhibiting normal flow and good graft recovery.

Certain limitations exist in case reports as they often focus on specific people or situations, which makes it challenging to apply their findings to larger groups. In addition, many case studies are retrospective and rely on past events or data, which may introduce recall bias or incomplete information. Conducting a multicenter real-world survey with a larger sample size would be instrumental in establishing the efficiency and safety of IA columns in a real-world setting.

## Conclusions

This case report concluded that desensitization using IA columns successfully achieves the targeted anti-B antibody titer, leading to good graft and recipient outcomes. The use of IA columns for desensitization in an ABOi KT patient proved effective, leading to a successful kidney transplant that was well-tolerated and devoid of postoperative adverse events. The positive outcome observed in this case adds to the increasing body of evidence validating the efficacy of IA columns in broadening the scope of potential kidney transplant recipients.
